# Nurse Faculty Enrichment and Competency Development in Oral-Systemic Health

**DOI:** 10.1155/2012/567058

**Published:** 2012-05-08

**Authors:** Maria C. Dolce

**Affiliations:** New York University College of Nursing, 726 Broadway, 10th Floor, New York, NY 10003, USA

## Abstract

Nurses are positioned to play a significant role in oral health promotion and disease prevention across the life cycle. Oral health has not been a high priority in nursing practice, and educating nurses about oral health has been inadequate particularly regarding the interrelationship between oral health and overall health. The first step for developing a nursing workforce with core competencies in oral health promotion and disease prevention is to prepare nurse faculty with the requisite knowledge, skills, attitudes, and best practices in oral-systemic health. The purpose of this paper is to present *Smiles for Life: A National Oral Health Curriculum* as a knowledge framework that nurse faculty can use for faculty enrichment and competency development in oral health across the life cycle. A variety of teaching-learning strategies and resources are provided to assist nurse faculty with integrating oral-systemic health into existing nursing curricula.

## 1. Introduction

 Nurses are positioned to play a significant role in oral health promotion and disease prevention across the life cycle, and in expanding access to preventive care services especially for vulnerable and underserved populations across health care settings [1–5]. Oral health promotion and preventive care services, such as basic risk assessments, oral health examinations, referrals, counseling, and anticipatory guidance, are well within the scope of professional nursing practice. Recognizing the significance of oral health in achieving overall health and well-being, nurses can empower individuals, families, and communities with oral health information and resources to support healthy choices regarding oral hygiene, diet, tobacco, and alcohol use.

 Historically, oral health has not been a high priority in nursing practice [1]. Education and training of nurses about basic oral health and oral-systemic health has been inadequate [5–7]. In 2007, a survey of academic deans and administrators from accredited dental schools in the United States and Canada found that only 2.3% of respondents strongly agreed that nurses and physicians in their health science center or academic campus were well educated about oral-systemic health [8]. The potential role of nurses in improving oral health outcomes across the life cycle and expanding access is contingent upon enhancing nursing curricula in undergraduate- and graduate-level programs and professional development programs for practicing nurses [1]. 

The initial step for developing a nursing workforce with core competencies in oral health promotion and disease prevention is to prepare nurse faculty with the requisite knowledge, skills, attitudes, and best practices in oral-systemic health. The purpose of this paper is to present *Smiles for Life: A National Oral Health Curriculum* [9] as a knowledge framework that nurse faculty can use for competency development in oral health across the life cycle. A variety of teaching-learning strategies and resources are provided to assist nurse faculty with integrating oral-systemic health into existing nursing curricula.

## 2. Materials and Methods

### 2.1. Smiles for Life: A National Oral Health Curriculum

*Smiles for Life* (*SFL*) is a comprehensive oral health curriculum developed by a national steering committee of physicians and dentists formed within the Society of Teachers of Family Medicine, and specially tailored for primary care clinicians and educators [9]. Since its launch in 2006, this online resource (http://www.smilesforlifeoralhealth.org/) has been accessed by individual users including physicians, nurses, physician assistants, and dental hygienists [9]. The curriculum has been adapted and implemented in medical schools and residency programs [9, 10]. *Smiles for Life *curriculum has received numerous endorsements from medical and nursing organizations, including American Academy of Family Physicians, American Academy of Pediatrics, Society of Teachers of Family Medicine, American Academy of Physician Assistants, Association of Faculties of Pediatric Nurse Practitioners, National Association of Pediatric Nurse Practitioners, and Gerontological Advanced Practice Nurses Association.

*Smiles for Life* is an evidence-based curriculum covering oral health across the life cycle. The curriculum is presented in an interactive, web-based format and consists of eight courses: (1) The Relationship of Oral to Systemic Health, (2) Child Oral Health, (3) Adult Oral Health, (4) Acute Dental Problems, (5) Oral Health and the Pregnant Patient, (6) Fluoride Varnish, (7) The Oral Examination, and (8) Geriatric Oral Health. *Smiles for Life* curriculum is approved for continuing nursing education by New York University College of Nursing's Center for Continuing Education in Nursing, an accredited provider of continuing nursing education by the American Nurses Credentialing Center's Commission on Accreditation. Each individual course is approved for 1.0 contact hour and is available free to individual users.

#### 2.1.1. Clinicians

 Individual clinicians can access the *SFL* curriculum and complete the online courses at their own pace. New users are advised to complete the registration information before launching a course. Each course will take approximately one hour to complete. Clinical cases corresponding to each course are included as optional learning activities. Each case will take approximately 10 minutes to complete. Clinicians are recommended to review the clinical cases before completing the post-assessment. Only registered users will be eligible to complete the post-assessment, obtain a certificate of completion, and receive continuing education credit.

#### 2.1.2. Educators

 A special link for educators is located on the *SFL* website homepage. Nurse educators can readily access educational materials for integration into existing nursing curricula and professional development programs. Each individual course is downloadable and includes course description, educational objectives, PowerPoint slides, presenter notes, clinical cases, test questions, instructional videos, and implementation guide. Additional *SFL* resources, described in the next section, are available for integration into clinical teaching and practice settings.

#### 2.1.3. Resources

*Smiles for Life* offers a multitude of educational and practice resources for educators and clinicians. Reference guides such as lab coat “pocket cards” can be downloaded to assist students and nurses in delivering patient care. For example, the *Adult Oral Health Pocket Card* provides clinicians with evidence-based recommendations for the prevention of periodontal disease (gum disease) and caries (tooth decay), and treatment of xerostomia (dry mouth) in adult patients. Patient education handouts and posters are available in English and other languages, including Spanish, Vietnamese, Cambodian, and Russian. The handouts can be printed and made available to patients, and the posters can be displayed in patient care areas such as waiting and examination rooms. Brief instructional videos are available on the website and can be downloaded into PowerPoint presentations. Instructional videos include lap-to-lap child oral exam, application of fluoride varnish, palpation of the temporomandibular joint, and palpation of the floor of the mouth. The *SFL* website also provides a selected annotated bibliography for clinicians and educators. Oral health topics include (a) engaging primary care medical providers in preventing dental disease, (b) diabetes and oral health, (c) prenatal/perinatal oral health, (d) cardiovascular disease and oral health, (e) seniors and oral health, and (f) other oral systemic health connections. The *SFL* website offers clinicians and educators direct links to peer-reviewed internet sites for oral health information and resources.

## 3. Results and Discussion

### 3.1. Knowledge Framework

 The *Smiles for Life* curriculum provides a knowledge framework for nurse faculty enrichment and competency development in oral health across the life cycle.  Table 1 outlines a strategy for nurse faculty to enhance nursing competencies in the domain of oral-systemic health. This strategy addresses the competency to integrate knowledge of the oral health impact on systemic health into professional nursing practice. Building upon the *SFL* knowledge framework, educational strategies and resources are identified for nurse faculty enrichment. Teaching-learning strategies are described for integration into existing nursing curricula.

#### 3.1.1. Content

 The first *SFL* course, *The Relationship of Oral to Systemic Health,* provides nurse faculty and students with a core knowledge base in oral-systemic health interactions. This foundational course addresses the linkage between oral health and systemic health, and discusses the role of clinicians in promoting oral health and preventing oral diseases. At the completion of the first course, nurse faculty and students will be able to (a) discuss the prevalence and sequelae of oral disease, (b) recognize the interrelationships between oral and systemic disease, and (c) highlight the role of professional nurses in promoting oral health. Most learners will take approximately one hour to complete the first course. It is recommended that learners complete the two optional clinical cases included in the oral-systemic health module, review the selected annotated bibliography for references addressing the association between periodontal disease and systemic diseases, and navigate the recommended oral health websites.

#### 3.1.2. Resources for Faculty Enrichment

 The US Surgeon General's landmark report released in 2000, *Oral Health in America*, is a good starting point for understanding the meaning of oral health and the link between oral health and overall health. The report described the mouth as a “mirror” of health and disease and emphasized that oral health encompasses a broader scope than healthy teeth [11]. Over a decade later, the 2011 Institute of Medicine's reports on oral health conveyed a consistent message about the importance of oral health to overall health and well-being, and the imperative to enhance the role of health professionals in oral health promotion and disease prevention [4, 5].

 Improving oral health is a national priority and a global challenge. *Healthy People 2020* outlined a national agenda with goals and objectives for improving health for all Americans and identified oral health as one of the leading health indicators [12]. The *Healthy People 2020* website (http://www.healthypeople.gov/2020/default.aspx) is an excellent resource for information about the oral health impact on overall health, oral health disparities in the United States, and evidence-based recommendations on oral health. The World Health Organization (WHO) established a strategic *Global Oral Health Programme *focused on developing global policies addressing oral health promotion and disease prevention [13]. The WHO website (http://www.who.int/oral_health/en/) is an important resource for strategies and approaches on oral health promotion and disease prevention. The WHO defined oral health as “a state of being free from chronic mouth and facial pain, oral and throat cancer, oral sores, birth defects such as cleft lip and palate, periodontal (gum) disease, tooth decay and tooth loss, and other diseases and disorders that affect the oral cavity. Risk factors for oral diseases include unhealthy diet, tobacco use, harmful alcohol use, and poor oral hygiene” [14]. This definition is useful in understanding that oral health is more than healthy teeth, oral care is greater than dental care, and oral diseases share the same risk factors as many significant noncommunicable diseases such as cardiovascular disease and cancer.

#### 3.1.3. Integrative Teaching-Learning Strategies

 Strategies that actively engage learners and integrate knowledge of oral-systemic health interactions are recommended for teaching nursing students about the oral-systemic health connection. A variety of effective teaching-learning methods can be used to integrate knowledge about oral-systemic health into professional nursing practice, such as clinical case presentations, clinical simulations, and unfolding case studies. Clinical case presentations are effective in illustrating the oral manifestations of systemic diseases or oral-systemic interactions. Clinical case examples may include patients with diabetes mellitus and periodontal disease, eating disorders and oral disease, sexually transmitted diseases including HIV/AIDS and oral manifestations, depression and oral impact of medications, and impact of cancer treatments (radiation and chemotherapy) on oral health. Clinical simulation scenarios can be designed to address the role of professional nurses in the assessment and monitoring of the oral health impact of systemic conditions and treatments. Case scenarios focused on oral health promotion and disease prevention can include counseling mothers about good oral hygiene during pregnancy, teaching children proper tooth brushing, and providing oral care to patients with disabilities or special needs. Nurse faculty can develop unfolding case studies to correlate patient's oral manifestations of systemic disease and pathophysiology, and to plan and evaluate interventions for an oral-systemic interaction. Unfolding case study examples may include patients with xerostomia related to HIV treatment, poor glycemic control associated with chronic periodontal disease, and poor nutritional status and edentulism. Additional teaching-learning strategies are outlined in Table 1 to assist nurse faculty with integrating knowledge of oral-systemic health into existing nursing curricula.

## 4. Conclusion

*Smiles for Life* is a comprehensive oral health curriculum for health professionals and serves as a knowledge framework for nurse faculty enrichment and competency development in oral health across the life cycle. Articulating the importance of oral health to overall general health is essential in promoting oral health and preventing oral disease. Nurse faculty and educators are called to develop a nursing workforce that understands oral-systemic health associations and demonstrates the core competencies of oral health promotion and disease prevention. To attain this goal, strategies and resources are recommended for nurse faculty enrichment. Teaching-learning strategies that actively engage nursing students about the oral-system connection will contribute to the integration of knowledge about oral-systemic interactions into professional nursing practice.

## Figures and Tables

**Table 1:** Framework for faculty enrichment and competency development in oral health.

Competency domain	Competency outcome	* Smiles for Life *curriculum	Faculty enrichment resources	Integrative teaching-learning strategies
			Read the following references:	
		Complete *SFL Course 1: The Relationship of Oral to Systemic Health *	US Department of Health and Human Services, Office of the Surgeon General (2000). *Oral health in America: A report of the surgeon general*. Rockville, MD: Author.Retrieved from http://www.surgeongeneral.gov/library/reports/oralhealth/ [11]	Incorporate *SFL Course 1* post-assessment questions in didactic teaching using audience response system
		Review optional clinical cases corresponding to oral-systemic health	Institute of Medicine (2011). *Advancing oral health in America*. Washington, DC: The National Academies Press. Retrieved from http://iom.edu/Reports/2011/Advancing-Oral-Health-in-America.aspx [4]	Assign students to complete *SFL Course 1* in preparation for a clinical simulation
Oral-systemic health interactions	Integrate knowledge of oral health impact on systemic health into professional nursing practice	Complete post-assessment and achieve passing score of 80% or higher	Institute of Medicine. (2011). *Improving access to oral health care for vulnerable and underserved populations*. Washington, DC: The National Academies Press. Retrieved from http://www.iom.edu/Reports/2011/Improving-Access-to-Oral-Health-Care-for-Vulnerable-and-Underserved-Populations.aspx [5]	Incorporate *SFL* patient education handouts in clinical or simulation experiences
		Review selected annotated oral health bibliography for references addressing associations between periodontal disease and diabetes mellitus, adverse pregnancy outcomes, cardiovascular disease	Navigate the following websites: *Healthy People 2020, Oral Health* at http://healthypeople.gov/2020/topicsobjectives2020/overview.aspx?topicid=32	Instruct students to use *SFL* pocket cards as a reference guide during clinical or simulation experiences
		Navigate recommended oral health websites	*World Health Organization Global Oral Health Programme* at http://www.who.int/oral_health/en/	Select a research article from *SFL* annotated bibliography and facilitate an evidence-based journal club discussion
